# A unified progressive fusion network with adaptive degradation prompts for multi-weather thermal infrared image restoration

**DOI:** 10.1371/journal.pone.0362272

**Published:** 2026-11-10

**Authors:** Yinxuan Xia, Shaoshuo Mu

**Affiliations:** 1 School of Media Engineering, Communication University of Zhejiang, Hangzhou, China; 2 Key Lab of Film and TV Media Technology of Zhejiang Province, Hangzhou, China; University of Electronic Science and Technology of China, CHINA

## Abstract

Thermal infrared (TIR) image restoration aims to recover clearer and more informative images from inputs degraded by unfavorable weather conditions. Current TIR restoration approaches, however, mainly focus on individual degradation categories, such as rain, snow, or haze, which limits their ability to address different weather degradation categories within a unified framework. To address this challenge, we introduce a TIR unified prompt-guided fusion network, an adaptive degradation-prompting framework designed for unified TIR image restoration under individually occurring rain, snow, or haze degradations. A weather-adaptive dynamic prompt module is first constructed, where a lightweight degradation predictor estimates the probability distribution over predefined degradation categories in TIR images and produces dynamic degradation prompts from a learnable prompt bank. The generated degradation prompts are then provided to the progressive fusion enhancement module and integrated through a prompt fusion strategy to guide feature enhancement and restoration, allowing the network to restore TIR images effectively under diverse weather conditions. To improve restoration quality across the evaluated degradation categories, we further design a joint loss function that combines Charbonnier reconstruction loss, Laplacian edge loss, and degradation-aware auxiliary constraints. Experimental results on the M3FD-based synthetic benchmark dataset demonstrate that the proposed framework achieves the best overall performance and competitive or superior performance across most evaluated degradation conditions, while effectively suppressing degradations caused by rain, snow, and haze. Moreover, the restored images achieve competitive performance in downstream object detection, further demonstrating the potential of the proposed framework for adverse-weather TIR perception. The source code is available at https://github.com/xia11998/TIR-UPF.

## 1. Introduction

Thermal infrared (TIR) imaging is widely used in unmanned aerial vehicle (UAV) remote sensing, video surveillance, autonomous driving, and other vision applications [1–3]. Unlike visible imaging, thermal infrared imaging does not depend on external illumination, enabling more robust perception under low-light, nighttime, and complex illumination conditions. However, TIR images in real-world environments remain susceptible to weather-related degradations, including rain, snow, and haze, which commonly reduce thermal contrast and weaken fine-grained structural details. These degradations consequently impair downstream tasks, such as object detection, recognition, and scene understanding [1,2,4]. Therefore, the proposed unified TIR image restoration strategy can serve as a preprocessing component for adverse-weather perception systems by improving the visibility of pedestrians, vehicles, and road structures in autonomous driving and intelligent transportation, while also enhancing target boundaries and thermal contrast for thermal surveillance, unmanned aerial sensing, mobile robotics, and search-and-rescue applications.

Existing research on image restoration under complex weather conditions can be generally divided into three directions: unified visible image restoration, infrared-assisted restoration and multimodal fusion, and thermal infrared image enhancement. For unified visible image restoration, TransWeather [5] employs weather-specific queries to handle multiple adverse weather conditions, while PromptIR [6] introduces learnable prompts to adapt restoration to different degradations. However, these methods are primarily designed for visible images and do not explicitly account for the thermal-radiation characteristics of TIR images. Infrared-assisted and multimodal approaches, such as VIFNet [7], CAWM-Mamba [8], and AWFusion [9], exploit complementary information between visible and infrared images for restoration or fusion, but generally rely on paired visible–infrared inputs. For TIR image restoration, Erlenbusch et al. [4] and T-SFANet [10] focus mainly on thermal infrared dehazing, while related studies have explored other thermal infrared restoration and enhancement tasks [11–13]. More recently, PPFN [14] introduces prompt fusion for infrared image enhancement. Nevertheless, these representative TIR methods either focus on specific restoration tasks or rely on predefined degradation-related prompt forms, leaving adaptive restoration of single TIR images across different weather degradation categories insufficiently explored. In contrast, the proposed method directly estimates the degradation-state distribution from a single degraded TIR image, generates an input-dependent dynamic degradation prompt, and progressively integrates it into restoration features, enabling a unified model to adapt to rain, snow, and haze without additional modality inputs or manual specification of the degradation type. In this study, unified restoration refers to handling individually occurring rain, snow, or haze degradations within a single model, rather than simultaneous combinations of these degradations. The main contributions of this study are summarized as follows:

- The proposed TIR-unified prompt-guided fusion (TIR-UPF) provides a unified framework for thermal infrared image restoration across individually occurring rain, snow, or haze degradations without requiring manual specification of the degradation type during inference.
- The weather-adaptive dynamic prompt (WDP) module and the progressive fusion enhancement (PFE) module are introduced to jointly improve restoration by estimating degradation-state distributions, producing dynamic degradation prompts, and adaptively modulating restoration features via prompt fusion.
- A joint loss function specifically designed for thermal infrared restoration is introduced. This loss integrates Charbonnier reconstruction loss, edge loss, and degradation-aware auxiliary constraints to enhance restoration quality, maintain target boundaries, and improve the discriminative ability of degradation prompt representations.
- Comprehensive experiments on the M3FD-based synthetic benchmark dataset demonstrate that TIR-UPF achieves the best overall performance and competitive or superior performance across most evaluated degradation conditions. Furthermore, downstream object detection experiments confirm its potential utility for robust thermal infrared perception in adverse weather conditions.

## 2. Related work

### 2.1 Visible image restoration under adverse weather conditions

Image restoration in adverse weather has traditionally been investigated mainly for visible images, covering representative tasks, such as deraining, desnowing, dehazing, denoising, and deblurring. Earlier approaches commonly depended on physical priors or task-specific networks to handle a single degradation type. Although these methods can obtain promising results under particular degradation conditions, their adaptability remains restricted when the degradation category is unknown, the degradation intensity changes, or multiple degradations occur simultaneously. To enhance the generalization capability of restoration models, unified image restoration has recently received growing attention. Zamir et al. [15] presented Restormer, an efficient transformer-based architecture for high-resolution image restoration, which has been extensively adopted as a strong backbone in later restoration tasks. Valanarasu et al. [5] proposed TransWeather, which uses a transformer architecture and a weather-query mechanism to restore images affected by diverse weather conditions. Li et al. [16] introduced AirNet to learn degradation-aware representations through contrastive learning, allowing the model to address unknown corruptions. Prompt-based and adaptive restoration methods, including PromptIR and AdaIR, further incorporate learnable prompts, frequency mining, and modulation mechanisms to improve all-in-one restoration performance [6,17]. Recent studies have also investigated visual in-context learning [18], vision-language degradation control [19], diffusion-based universal restoration [20], latent-diffusion-based all-in-one restoration [21], and instruction-following restoration [22]. These methods show that degradation-aware representations and conditional modulation mechanisms are effective for improving model generalization under complex degradation conditions. However, they are primarily developed for visible images, where degradation modeling generally depends on visual cues, such as color, texture, and luminance distributions. This dependence makes direct adaptation to thermal infrared images challenging, as such images mainly represent thermal radiation distributions.

### 2.2 Thermal infrared image restoration under adverse weather conditions

TIR images are generated from thermal radiation differences between targets and backgrounds and are less influenced by ambient illumination, making them particularly useful for nighttime, low-light, and complex illumination scenarios. However, thermal infrared imaging is not entirely resistant to weather-induced degradation. Studies by Bijelic et al. [1] and Rivera Velázquez et al. [2] have shown that under adverse weather conditions, particularly dense fog, TIR images can still experience decreased thermal contrast, weakened edges, and loss of target details, which further reduces object detection and recognition performance in autonomous driving, video surveillance, and unmanned systems. The multimodal adverse-weather perception study conducted by Bijelic et al. [1] shows that non-visible sensors, such as thermal infrared cameras, can offer complementary information for perception systems in complex weather conditions. Rivera Velázquez et al. [2] further reported that fog density, sensor field of view, and imaging conditions can substantially influence the quality of thermal infrared images. Recent studies have started to investigate the restoration of degraded thermal infrared images themselves. For instance, Erlenbusch et al. [4] examined thermal infrared single-image dehazing using the atmospheric scattering model, while Li et al. [10] introduced T-SFANet to enhance thermal infrared dehazing through spatial and frequency-domain attention. In addition, research on thermal infrared super-resolution, destriping, and deblurring shows that structure preservation and degradation modeling designed for the characteristics of thermal infrared imaging are critical for improving image quality [3,11,12,23,24]. These studies have contributed to the development of thermal infrared image restoration and enhancement. However, representative thermal infrared restoration methods still primarily focus on specific degradation scenarios or enhancement tasks, while unified modeling of multiple weather degradations, including rain, snow, and haze, remains relatively underexplored.

### 2.3 Infrared-assisted restoration and multimodal fusion

Another relevant line of research employs thermal infrared images as a complementary modality to improve visible image restoration and perception under adverse weather conditions. Xie and Jin [25] utilized the relatively stable contour cues provided by TIR images in hazy environments to introduce structural constraints for visible image dehazing. Infrared-visible fusion approaches further integrate cross-modal information through multi-scale feature extraction, attention-based mechanisms, collaborative learning, or frequency-aware cross-attention, which improves scene representation and object detection performance in challenging weather conditions [7,26,27,28]. Liu et al. [29] also introduced the M3FD benchmark, which has become an important dataset for infrared-visible image fusion and detection studies. More recent all-weather multimodal fusion frameworks, such as CAWM-Mamba [8] and AWFusion [9], have attempted to integrate multi-weather restoration and infrared-visible image fusion within unified architectures. These studies highlight the strong potential of thermal infrared information for adverse-weather perception. However, their primary focus is typically on multimodal fusion or visible image restoration, and they often rely on synchronously acquired visible-infrared image pairs. By comparison, the adaptive restoration of single TIR images degraded by weather effects has received limited attention. In particular, only a few methods can automatically adapt the restoration process according to the degradation state of the input image without requiring additional modality inputs.

### 2.4 Prompt learning for degradation-adaptive restoration

Prompt learning has recently been applied to low-level vision restoration tasks, offering a new conditional modulation strategy for unified image restoration. Potlapalli et al. [6] introduced PromptIR, which represents degradation-related information with learnable prompts and embeds them into the restoration network, allowing the model to handle multiple restoration tasks under unknown degradation conditions. Later studies on multi-degradation restoration further investigated approaches, such as component knowledge modeling, dynamic routing, vision-language degradation representation, and frequency modulation to enhance degradation perception and restoration performance under complex degradation conditions [17,19,30,31]. For thermal infrared images, Liu et al. [14] proposed PPFN, which uses prompt fusion mechanisms for infrared image enhancement and demonstrates the potential of prompt modulation in infrared degradation restoration. However, current prompt-based methods commonly depend on predefined degradation types, fixed prompt structures, or degradation features obtained from visible images, while giving insufficient attention to thermal radiation distributions, target boundaries, and local thermal gradients in TIR images. When the input degradation type is uncertain or the degradation severity changes considerably, fixed prompts often cannot adequately correspond to the actual degradation state. Therefore, the development of a degradation-aware dynamic prompt mechanism for single thermal infrared images, together with its integration into a progressive restoration network, remains insufficiently studied in multi-weather thermal infrared image restoration. In contrast, TIR-UPF focuses on single-TIR-image restoration and combines input-dependent degradation prediction with dynamic prompt generation and progressive prompt fusion within a unified restoration framework.

## 3. Proposed method

### 3.1 Method overview

To address uncertainty in degradation types within single thermal infrared images under weather conditions, including rain, snow, and haze, we propose TIR-UPF, an adaptive degradation-aware progressive prompt fusion network for thermal infrared image restoration. The proposed model restores images through a degradation-aware progressive pipeline composed of a WDP module and multiple PFE modules, as illustrated in Fig 1. The WDP module first estimates the degradation-category distribution of the input TIR image and generates the dynamic degradation prompt Pdyn. This dynamic prompt is then integrated into the PFE module through prompt fusion to adaptively guide feature restoration under different degradation conditions. The entire network progressively refines the restored image through three PFE stages and is optimized using a joint loss function for reconstruction, edge preservation, and degradation-aware supervision.

**Fig 1:**
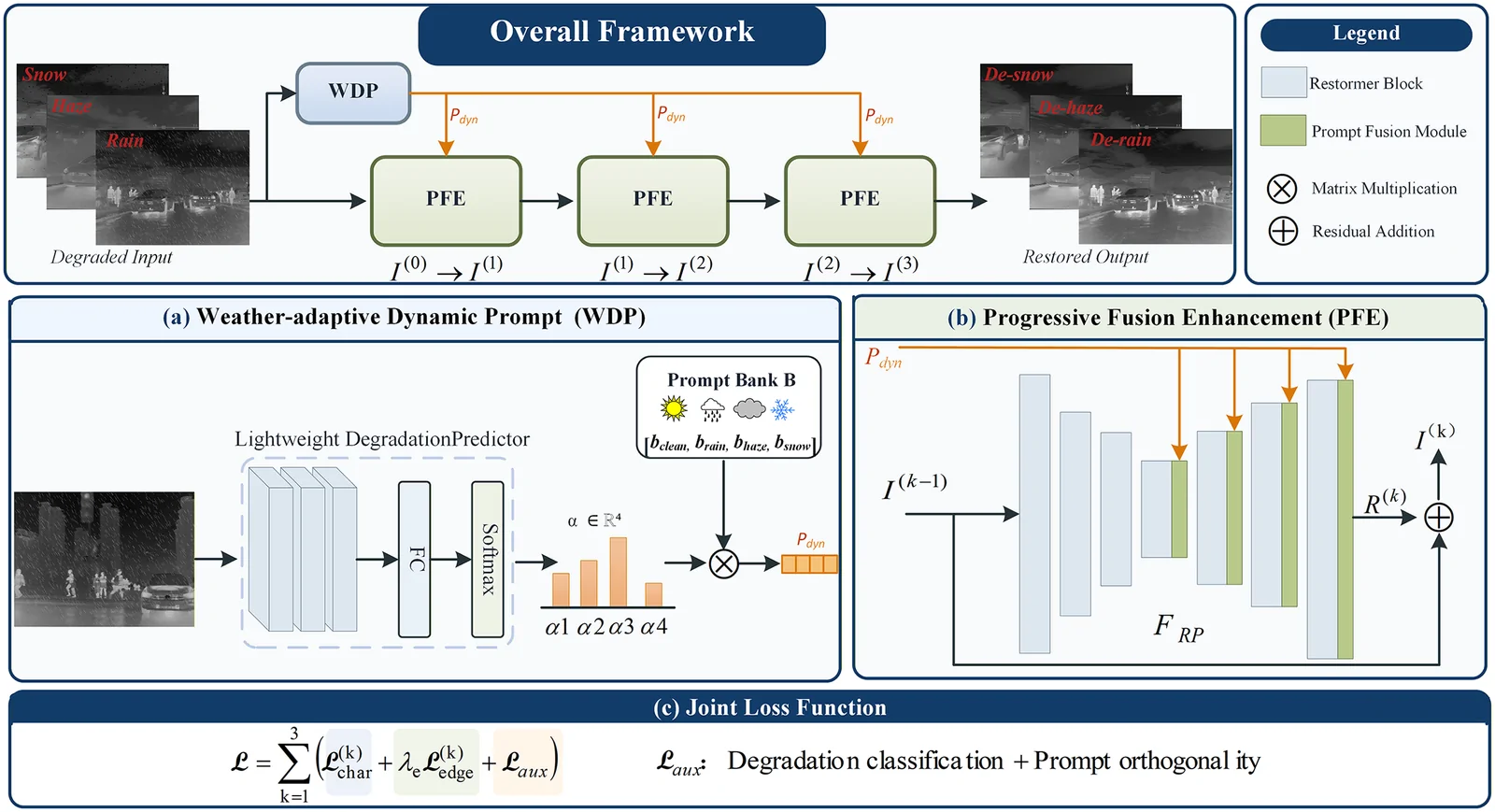
Overall architecture of TIR-UPF. The framework comprises three main components: WDP, PFE, and joint loss optimization. In WDP, the degradation predictor produces the dynamic prompt P_*dyn*_, whereas PFE integrates P_*dyn*_ to perform thermal infrared image restoration. The example infrared source images (files: 0011.png, 0018.png, 0021.png, and 0006.png) were obtained from the M3FD_Fusion_train archive of the Figshare dataset by Huang [32], which is licensed under CC BY 4.0. The degraded/restored example panels were generated by the authors and are presented for illustrative purposes only; all annotations and the overall schematic were created by the authors.

### 3.2 Weather-adaptive dynamic prompt (WDP)

TIR imaging can be degraded by weather conditions, including rain, snow, and haze, while the degradation state may also vary or remain uncertain in complex weather scenarios. When the model depends solely on fixed prompts or manually defined degradation categories, adapting to changes in the degradation state of the input image becomes difficult. To address this problem, we introduce a lightweight degradation predictor and a learnable prompt bank, and design a WDP module that estimates the degradation-category distribution from the input image and generates corresponding dynamic degradation prompts.

The degradation predictor consists of two 3 × 3 convolutional layers with a stride of 2 and a padding of 1, which map the input from 1 to 32 and then 64 channels, with each convolution followed by a ReLU activation. Global average pooling is then applied, followed by a linear layer that produces four degradation-category logits. A softmax operation converts the logits into the degradation probability vector $\alpha \in R^{\text{1}\times \text{4}}$. Based on the predicted distribution, a prompt bank is constructed to define a learnable prompt library for different degradation states, including clean, haze, rain, and snow, as formulated below:


$$\begin{array}{c} B={\left[b_{\text{1}},b_{\text{2}},b_{\text{3}},b_{\text{4}}\right]}^{T} \end{array}$$ (1)

where $b_{\text{i}}$ represents the learnable prompt embedding corresponding to the *i*-th degradation state. Specifically, the prompt embeddings $b_{\text{1}},b_{\text{2}},b_{\text{3}},$ and $b_{\text{4}}$ correspond to the clean, haze, rain, and snow states, respectively. The degradation probability vector α is subsequently used to aggregate the prompt bank and generate the dynamic degradation prompt ${\text{P}}_{dyn}$. In this work, each learnable degradation prompt is represented by a 64-dimensional vector. Accordingly, the prompt bank contains four prompt vectors for the clean, haze, rain, and snow categories, forming a 4 × 64 learnable prompt matrix. The dynamically generated prompt therefore has a dimensionality of 64.


$$\begin{array}{c} P_{dyn}=\alpha ⊗B \end{array}$$ (2)

where ⊗ denotes the weighted aggregation of the degradation probability vector α and the prompt bank B. This design allows the model to represent the degradation state of the input image within a predefined degradation-category space through continuous weights. When α approaches a one-hot vector, the dynamic degradation prompt ${\text{P}}_{dyn}$ becomes more similar to the prompt prototype of a specific degradation type. When the distribution of α spans multiple degradation categories, the dynamic degradation prompt ${\text{P}}_{dyn}$ can reflect uncertainty among these categories through continuous weighting. Since the predictor is supervised using one-hot labels for the four predefined categories, the resulting soft distribution is intended to capture uncertainty within this predefined degradation space, rather than explicitly modeling unseen mixed degradation patterns. During training, the degradation classification loss directly supervises the degradation predictor, while the restoration loss jointly optimizes the degradation predictor and prompt-bank parameters through the dynamic prompt generation path. During inference, the corresponding dynamic prompt can be generated using only the input image.

### 3.3 Progressive fusion enhancement (PFE)

We adopt Restormer as the foundational restoration backbone because its architecture can model multi-scale feature representations and long-range dependencies, making it appropriate for feature reconstruction in image restoration tasks [15]. Based on its hierarchical encoder-decoder structure, we further design a progressive prompt fusion mechanism, in which the weather-adaptive dynamic degradation prompt ${\text{P}}_{dyn}$ is incorporated to provide continuous conditional modulation and progressive refinement within the shared backbone. As illustrated in Fig 1(b), a seven-layer PFE module is constructed using Restormer as the basis. Specifically, the prompt fusion mechanism is embedded in the upsampling layers. To project the prompt into the model feature space and improve its adaptability to different degradation conditions, the prompt fusion mechanism calculates two channel-wise modulation parameters with appropriate dimensions through a linear layer.


$$\begin{array}{c} \gamma^{m},\beta^{m}=\phi_{m}\left(P_{dyn}\right) \end{array}$$ (3)

where $\varphi_{m}$ represents the linear layer in the *m*-th prompt fusion mechanism, and m serves as the index of the prompt fusion mechanism. The terms γ and β represent the modulation parameters related to image degradation and degradation scenarios, respectively. Subsequently, in accordance with the principle of PPFN [14], the feature maps are modulated using the derived modulation parameters, as formulated below:


$$\begin{array}{c} \overset{\sim }{F}=F+\gamma^{m}⊙F+\beta^{m} \end{array}$$ (4)

where the feature map before modulation is denoted by F, whereas the fused feature obtained after the prompt fusion mechanism is denoted by \stackrel F. The channel-wise multiplication operator is represented by ⊙. This residual affine modulation incorporates conditional information associated with the restoration stage and degradation state while maintaining the feature distribution of the backbone. After multi-layer prompt fusion modulation, each PFE module introduces a residual connection to produce the module output:


$$\begin{array}{c} R^{\left(k\right)}=F_{RP}\left(I^{\left(k-1\right)},P_{dyn}\right) \end{array}$$ (5)

$$\begin{array}{c} I^{\left(k\right)}=I^{\left(k-1\right)}+R^{\left(k\right)},k=1,2,3 \end{array}$$ (6)

where the index *k* indicates the *k*-th PFE module, and $F_{\text{RP}}(\cdot )$ represents Restormer integrated with the prompt fusion mechanism. The output of $R^{\left(k\right)}$ is denoted by $F_{\text{RP}}(\cdot )$, whereas $I^{\left(k-\text{1}\right)}$ and $I^{\left(k\right)}$ correspond to the input and output of the *k*-th PFE module, respectively. By cascading multiple PFE modules, the overall model performs continuous progressive optimization, enabling the restoration process to transition from global recovery to local detail refinement.

### 3.4 Joint loss function

TIR restoration tasks typically use reconstruction losses to enforce consistency between restored outputs and reference images [3,14]. However, dependence on pixel-level reconstruction loss alone often produces overly smoothed edge regions in the restored images, which makes preserving fine structural details in thermal infrared images challenging. Since the proposed model incorporates a degradation predictor, the accuracy of the dynamic prompt ${\text{P}}_{dyn}$ may be reduced when the degradation prediction branch is not explicitly supervised. To address these problems, we formulate a joint optimization objective composed of reconstruction loss, edge loss, and degradation-aware constraints, as follows:


$$\begin{array}{c} L=\sum_{k=1}^{\text{3}}\left(L_{char}^{\left(k\right)}+\lambda_{e}L_{edge}^{\left(k\right)}\right)+3L_{aux} \end{array}$$ (7)

where $\lambda_{\text{e}}$ represents the weighting coefficient for the edge loss. As $L_{aux}$ is not tied to any specific restoration stage, it is included in the total loss function as a global auxiliary constraint. The three-stage cumulative supervision design follows the multi-stage progressive restoration strategy of MPRNet [33] and aligns with the three-stage architecture defined in Equation (6).

**Reconstruction loss.** The Charbonnier loss [34] is adopted to enforce consistency between the restored output at each stage and its corresponding supervision target.


$$\begin{array}{c} L_{char}^{\left(k\right)}=\frac{\text{1}}{N}\sum_{i=1}^{N}\sqrt{{\left(I_{i}^{\left(k\right)}-T_{i}^{\left(k\right)}\right)}^{\text{2}}+\varepsilon^{\text{2}}} \end{array}$$ (8)

where the restored result at the *k*-th stage is denoted by $I^{\left(k\right)}$, and the corresponding supervision target is represented by $T^{\left(k\right)}$. The *i*-th pixel values of the restored image and its corresponding supervision target at the *k*-th stage are denoted by $I_{i}^{\left(k\right)}$ and $T_{i}^{\left(k\right)}$, respectively. The total number of image pixels is denoted by *N*, while ε is a smoothing constant introduced to enhance the stability of the loss function and is set to $\text{1}\times {\text{10}}^{-\text{3}}$ in this work. For directly degraded samples, the supervision target is defined as $T^{\left(k\right)}=I^{c}$. Compared with the standard $L_{\text{1}}$ loss, the Charbonnier loss offers better differentiability as the residual approaches zero and shows greater robustness to pixels with large errors.

**Edge loss.** To improve the ability of the restored images to preserve structural information, we further incorporate an edge loss based on Laplacian responses.


$$\begin{array}{c} L_{edge}^{\left(k\right)}=L_{char}\left(\Delta \left(I^{\left(k\right)}\right),\Delta \left(T^{\left(k\right)}\right)\right) \end{array}$$ (9)

where the Laplacian operator is denoted by ∆; this loss is equivalent to applying the Charbonnier loss to the Laplacian edge responses of the restored image and the supervision target, thereby enforcing consistency in edge structures and target contours. For the weight configuration, we follow the commonly adopted edge-constraint settings in [33,35] and set $\lambda_{e}=\text{0}.\text{05}$.

**Degradation-aware auxiliary loss.** To improve the sensitivity of the degradation predictor to the degradation state of the input and enhance the discriminability among different degradation prompt prototypes, we introduce a degradation-aware auxiliary loss *L*_aux_. Specifically, *L*_aux_ consists of a degradation-aware cross-entropy classification term *L*_cls_ and a prompt orthogonality regularization term *L*_ortho_, corresponding to the first and second terms in Equation (10), respectively.


$$\begin{array}{c} L_{\text{aux}}=-\lambda_{\text{c}}\sum_{j=1}^{K}y_{i}log\alpha_{j}+\frac{\lambda_{\text{0}}}{K^{\text{2}}}{\|BB^{T}-I_{k}\|}_{F}^{\text{2}} \end{array}$$ (10)

The number of degradation categories is denoted by *K*, which is set to K=4 in this work and corresponds to the clean, haze, rain, and snow categories. The one-hot label of the ground-truth degradation category is denoted by $y_{j}$, whereas the probability of the *j*-th degradation category predicted by the degradation predictor after the softmax operation is represented by $\alpha_{j}$. The learnable prompt bank defined in Section 3.2 is denoted by *B*, the *K*-dimensional identity matrix is represented by $I_{k}$, and the Frobenius norm is denoted by ‖·‖.

In Equation (10), the cross-entropy term $-\lambda_{c}\sum_{j=1}^{k}y_{j}log\alpha_{j}$ supervises the degradation predictor by enforcing consistency between the probability vector α and the ground-truth degradation label, thereby providing reliable weights for the dynamic prompt ${\text{P}}_{dyn}$. The orthogonal term $\frac{\lambda_{o}}{K^{\text{2}}}{\|BB^{T}-I_{k}\|}_{F}^{\text{2}}$ follows the concept of soft orthogonal regularization, which encourages the Gram matrix to approximate an identity matrix [36], and employs $BB^{T}$ to represent correlations among different degradation prototype prompts. This constraint promotes discriminability among different degradation prototype prompts, prevents multiple prompt vectors from converging toward similar representations, and improves the ability of dynamic prompts to represent different weather-induced degradations. In this work, the empirical settings are $\lambda_{c}=\;\text{0}.\text{1},\;\lambda_{o}=\;\text{0}.\text{01}$.

## 4. Experimental results

### 4.1 Experimental settings

#### 4.1.1 Dataset construction and weather-induced degradation models.

Existing thermal infrared datasets generally lack paired clear and weather-degraded images [4]. Therefore, we use the thermal infrared images from M3FD [29] to construct paired restoration data, following synthetic degradation strategies commonly adopted in RESIDE [37] and FoggyCityscapes [38]. The 4,200 original images are divided at the original-image level into mutually exclusive training, validation, and test sets containing 2,940, 420, and 840 images, respectively. The validation set is used for checkpoint selection, whereas the test set is reserved for final evaluation. The partitioning is completed before degradation generation, and no content-identical or near-identical source images occur across the three subsets. After degradation generation, the 840-image test set comprises 93 clean, 239 haze, 244 rain, and 264 snow images. A four-category dataset consisting of clean, haze, rain, and snow images is constructed, with sampling proportions of 10%, 30%, 30%, and 30%, respectively. The clean category is used not only for degradation classification but also for restoration training and evaluation. During training, clean images are used as both the network inputs and their corresponding restoration targets. During evaluation, the 93 clean test images are also passed through the restoration models and are included in the aggregate evaluation over all 840 test samples. This distribution, adopted as a practical dataset-construction strategy rather than selected through a dedicated sampling-ratio sensitivity study, places greater emphasis on adverse-weather restoration while retaining a proportion of clean samples to represent non-degraded conditions.

From the perspective of a forward problem, the degraded thermal infrared image is represented as


$$\begin{array}{c} I_{d}=D_{k}(I_{clean};\theta_{k}),k\in \left\{rain,haze,snow\right\} \end{array}$$ (11)

where *D*_*k*_ denotes the degradation transformation and *θ*_*k*_ represents its parameters. Rain degradation is modeled by overlaying randomly distributed line-shaped streaks, followed by spatial smoothing and multiplicative intensity attenuation to simulate rain interference and thermal contrast reduction [1]. Haze degradation is generated using the atmospheric scattering model [37]:


$$\begin{array}{c} I_{haze}=clip\left[tI_{clean}+A\left(1-t\right),0,255\right] \end{array}$$ (12)

where *t* is the transmission coefficient and *A* denotes the atmospheric thermal-radiation value. Snow degradation is simulated by generating randomly distributed low-temperature circular spots, applying Gaussian smoothing to approximate thermal diffusion, and subtracting the resulting cold-spot layer from the original image [1,2].

All weather-degraded images used in this study are synthetically generated from the original M3FD thermal infrared images, and the resulting dataset is referred to as the M3FD-based synthetic benchmark. Specifically, the benchmark considers rain, snow, and haze as separate degradation categories, with only one weather degradation operator applied to each degraded image. For each image, the randomized degradation parameters are sampled independently. For rain degradation, the number of streaks is set to ⌊HW/600⌋, where H and W denote the image height and width, respectively. The starting positions of the streaks are sampled uniformly over the valid image area. The streak length and width are set to 15 pixels and 1 pixel, respectively, the horizontal slant offset is randomly sampled from [−10,10] pixels, the streak intensity is set to 200, a box blur with a 2 × 2 kernel is applied, and the intensity attenuation coefficient is set to 0.8. For haze degradation, the transmission coefficient and atmospheric thermal-radiation value are independently sampled as t ~ U(0.4,0.7) and A ~ U(80,120), respectively. For snow degradation, 300–1,000 circular cold spots are generated at random positions, with radii of 1–3 pixels and intensities sampled from U(30,80), followed by Gaussian smoothing with a 3 × 3 kernel. The resulting dataset contains degraded inputs, corresponding clean targets, and degradation-category labels.

#### 4.1.2 Training details.

In this study, all experiments are implemented in PyTorch and performed on an NVIDIA GeForce RTX 5070 Ti GPU. During the training of TIR-UPF, the input images are randomly cropped into 48 × 48 patches, and the batch size is set to 16. Random horizontal flipping is used for data augmentation. The Adam optimizer is adopted, with the initial learning rate of the restoration backbone set to 8 × 10^−5^. The learning rates for the degradation predictor and prompt-bank parameters are separately set to 1 × 10^−4^ to accelerate the early convergence of the degradation-aware branch. The model is trained for 300 epochs, using a linear warm-up strategy during the first three epochs, followed by cosine annealing to reduce the learning rate to 1 × 10^−6^.

All competing methods were reproduced using their official implementations released by the original authors. The original network architectures were retained, and all methods were retrained on the same training split and evaluated on the same test split. The training configurations of TIR-UPF and the competing methods are summarized in Table 1. For TIR-UPF, the learning rates of the restoration backbone and the degradation-aware branch were set to 8e-5 and 1e-4, respectively. For Restormer, the crop size and batch size were adjusted to 160 × 160 and 3, respectively, according to the available GPU memory. No independent hyperparameter tuning was performed for the competing methods.

**Table 1:** Training configurations of TIR-UPF and the competing methods.

Method	Epochs	Optimizer	LR	Crop	Batch
Restormer	300K iters	AdamW	3e-4	160 × 160	3
AirNet	1000	Adam	1e-3	128 × 128	5
PromptIR	120	AdamW	2e-4	128 × 128	8
AdaIR	150	AdamW	2e-4	128 × 128	8
AWFusion	200	Adam	1e-4	96 × 96	1
TIR-UPF	300	Adam	8e-5/1e-4	48 × 48	16

### 4.2 Comparisons with state-of-the-art

To verify the effectiveness of the proposed algorithm, we compare it with Restormer [15] (CVPR 2022), AirNet [16] (CVPR 2022), PromptIR [6] (NeurIPS 2023), AdaIR [17] (ICLR 2025), and AWFusion [9] (Information Fusion 2026). All comparison methods are retrained on the training set constructed in this study and evaluated on the same test set. PSNR, SSIM, and LPIPS are used as evaluation metrics. Higher PSNR and SSIM values reflect better reconstruction quality, whereas lower LPIPS values indicate better perceptual quality. For LPIPS evaluation, single-channel TIR images are converted to three-channel images by channel replication before being fed into the PyIQA LPIPS-v0.1 metric with an AlexNet backbone.

#### 4.2.1 Visual evaluation.

Figs 2–4 present the visual restoration results of different methods under rain, snow, and haze degradation scenarios, respectively. Existing methods can reduce weather-induced degradation to a certain extent; however, they still exhibit residual interference, limited local contrast, or insufficient recovery of structural details in severely degraded regions.

**Fig 2:**
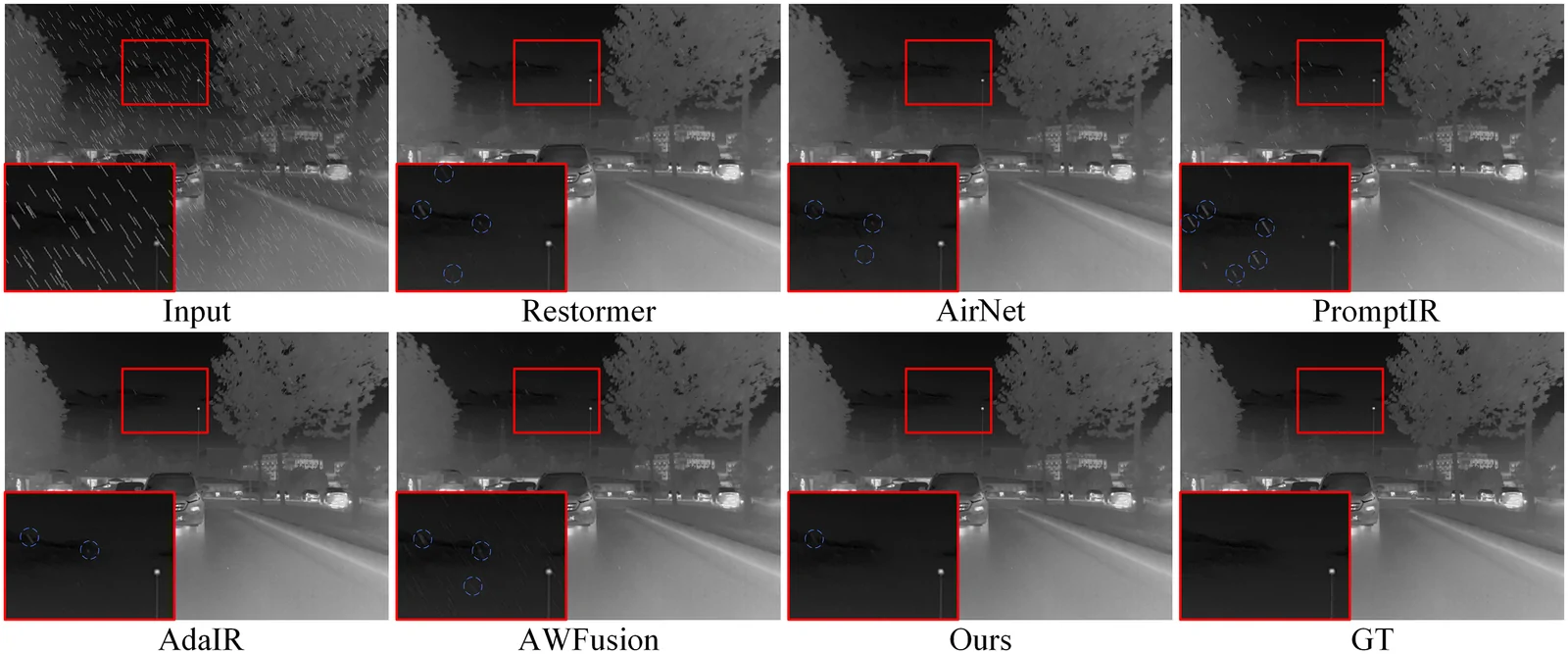
Visual comparison of different methods in the rain degradation scenario. The clean infrared source image (file: 0037.png) was obtained from the M3FD_Fusion_train archive of the Figshare dataset by Huang [32], which is licensed under CC BY 4.0. The synthetic rain degradation and all restoration results were generated by the authors.

**Fig 3:**
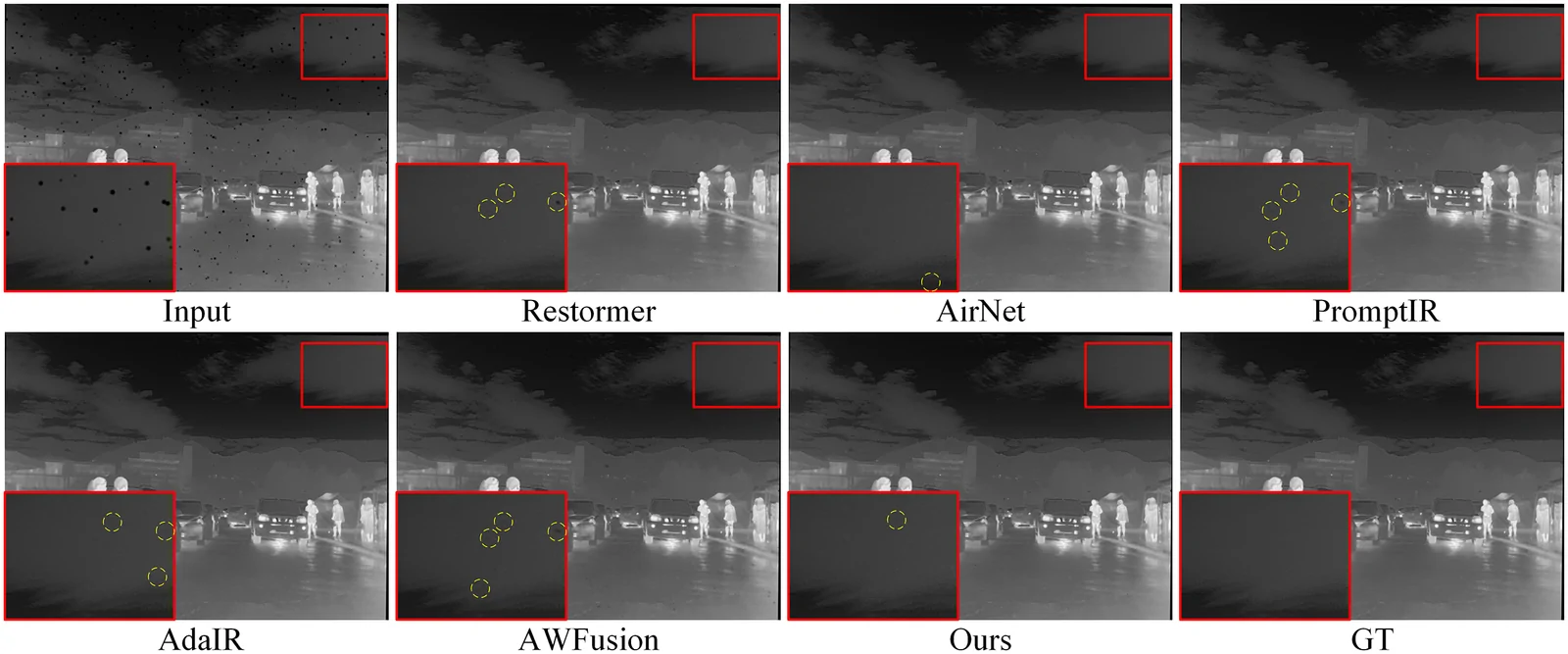
Visual comparison of different methods in the snow degradation scenario. The clean infrared source image (file: 0012.png) was obtained from the M3FD_Fusion_train archive of the Figshare dataset by Huang [32], which is licensed under CC BY 4.0. The synthetic snow degradation and all restoration results were generated by the authors.

**Fig 4:**
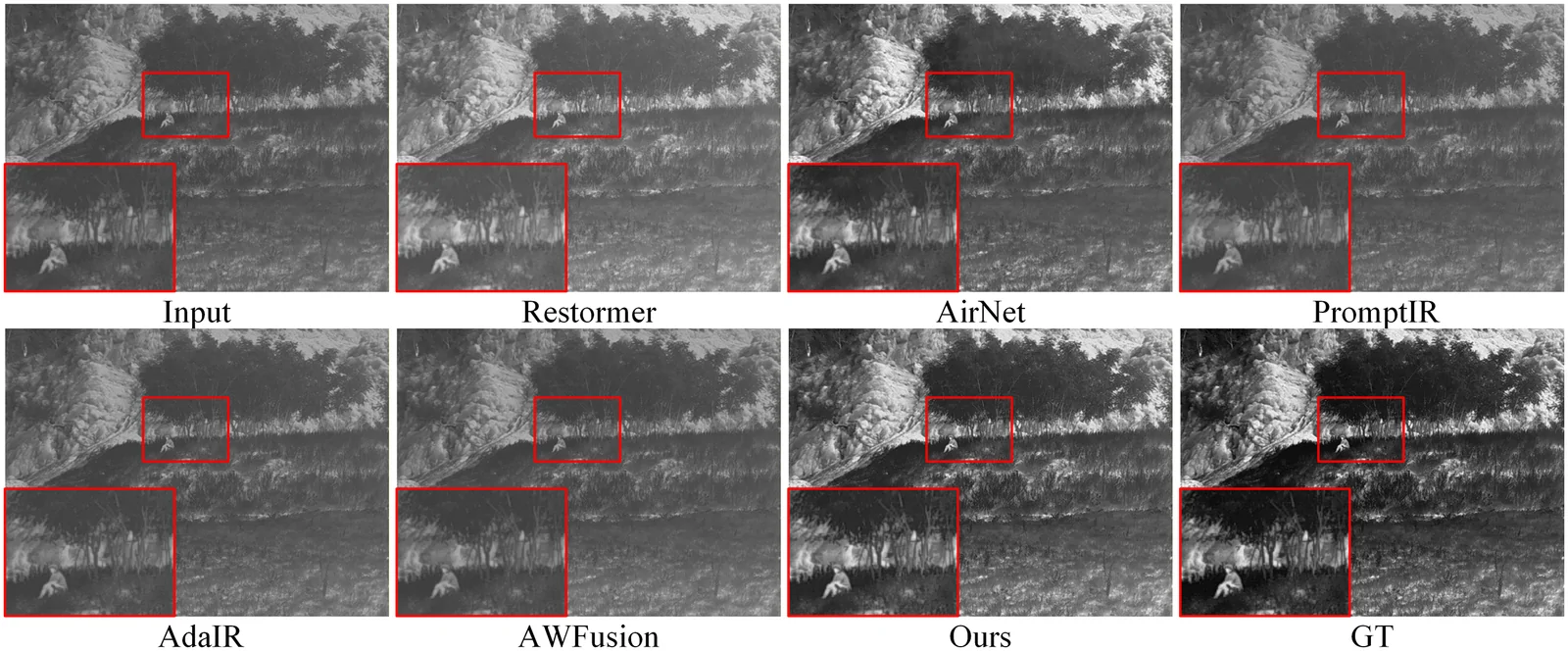
Visual comparison of different methods in the haze degradation scenario. The clean infrared source image (file: 0050.png) was obtained from the M3FD_Fusion_train archive of the Figshare dataset by Huang [32], which is licensed under CC BY 4.0. The synthetic haze degradation and all restoration results were generated by the authors.

As shown in Fig 2, Restormer and PromptIR still preserve slight rain streaks in several regions, whereas AirNet and AdaIR generate relatively smooth outputs with weakened target boundaries and road textures. In comparison, the proposed method effectively removes line-shaped rain streaks while maintaining target contours.

As shown in Fig 3, snow residues and local cold-spot interference remain visible in the outputs of several comparison methods. AWFusion also preserves local degradation residues in snow-affected regions. In comparison, the proposed method substantially reduces cold-spot interference and more effectively maintains structural details.

As shown in Fig 4, haze degradation leads to clear thermal contrast attenuation and weakens the hierarchical structure of the scene. Existing methods improve visual quality to a certain degree, but some results still show inadequate contrast recovery. The proposed method restores clearer thermal contrast and more complete scene structures. Overall, the proposed method obtains more stable restoration results under rain, snow, and haze degradations. These results indicate that TIR-UPF can effectively adapt to different degradation conditions and confirm the effectiveness of dynamic degradation prompts in unified thermal infrared image restoration.

#### 4.2.2 Quantitative evaluation.

Table 2 presents the quantitative results of different methods under rain, snow, and haze degradations, as well as across all test samples. The aggregate PSNR, SSIM, and LPIPS values are computed by directly averaging the per-image metrics over all 840 test images, rather than by macro-averaging the results across degradation categories. The all-sample results include the 93 clean inputs, which are also passed through the restoration network during evaluation. On the overall test set, TIR-UPF achieves the best performance in PSNR, SSIM, and LPIPS, with values of 39.3395 dB, 0.9692, and 0.0502, respectively. These results show that the proposed method achieves the best aggregate performance on the complete test set.

**Table 2:** Quantitative comparison under rain, snow, and haze degradations and across all test samples. Values are presented in the order of PSNR / SSIM / LPIPS. The best results are shown in bold, and the second-best results are underlined. N/A indicates that the PSNR of degraded inputs across all test samples is not reported because the degraded input is identical to the ground truth for clean images.

	Rain	Snow	Haze	All test samples
**Method**	**PSNR ↑ / SSIM ↑ / LPIPS↓**	**PSNR ↑ / SSIM ↑ / LPIPS↓**	**PSNR ↑ / SSIM ↑ / LPIPS↓**	**PSNR ↑ / SSIM ↑ / LPIPS↓**
Degraded	22.1918 / 0.7767 / 0.5820	34.5331 / 0.9580 / 0.3892	21.3736 / 0.8972 / 0.1621	N/A / 0.8927 / 0.3375
Restormer	37.8228 / 0.9665 / 0.1235	45.8420 / 0.9923 / 0.0486	22.1664 / 0.9079 / 0.1362	37.3391 / 0.9614 / 0.0902
AirNet	36.1955 / 0.9391 / 0.0934	47.1681 / 0.9932 / **0.0088**	26.3213 / 0.9318 / 0.0807	36.7522 / 0.9605 / 0.0552
PromptIR	35.7776 / 0.9473 / 0.1783	47.7712 / 0.9957 / 0.0186	22.6605 / 0.9129 / 0.1244	38.2316 / 0.9586 / 0.0931
AdaIR	34.0651 / 0.9338 / 0.0558	46.9593 / 0.9955 / 0.0106	21.8831 / 0.9051 / 0.1480	36.4114 / 0.9521 / 0.0614
AWFusion	37.5319 / 0.9603 / 0.1412	39.3230 / 0.9840 / 0.0580	22.0484 / 0.9045 / 0.1517	33.8905 / 0.9552 / 0.1036
Proposed	**38.4019** / **0.9726** / **0.0556**	**48.1167** / **0.9960** / 0.0210	**29.2031** / **0.9618** / **0.0361**	**39.3395** / **0.9692** / **0.0502**

Across different weather conditions, the compared methods show varying performance for different degradation types. In rainy scenarios, the proposed model obtains the best PSNR, SSIM, and LPIPS results, reaching 38.4019 dB, 0.9726, and 0.0556, respectively. This indicates that the proposed method can effectively suppress rain streaks while maintaining structural information and local details in TIR images. Under snow degradation, the proposed model achieves the highest PSNR and SSIM values of 48.1167 dB and 0.9960, respectively, while PromptIR obtains the second-best SSIM value of 0.9957. However, the proposed model records an LPIPS value of 0.0210 in snow scenarios, which is not the best result and remains inferior to the perceptual performance of AirNet, AdaIR, and PromptIR. This suggests that perceptual detail restoration under snow degradation still has potential for further improvement. In haze scenarios, the proposed model achieves the best PSNR, SSIM, and LPIPS results, with values of 29.2031 dB, 0.9618, and 0.0361, respectively. These results demonstrate that the proposed method has strong contrast recovery and perceptual restoration capability for low-contrast thermal infrared images degraded by haze.

Overall, the proposed model exhibits competitive performance across the three weather degradation types, with particularly evident advantages in rainy and hazy scenarios. Together with the overall results in Table 2, the proposed method maintains consistent metric performance under rain, snow, and haze degradations, indicating its adaptability across individually occurring weather degradation categories, rather than simultaneous combinations of these degradations.

### 4.3 Ablation study

To assess the contribution of each component, we conduct ablation experiments on dynamic prompt generation and fusion, the progressive PFE structure, and the joint loss function. All experiments use the same training configuration, with only the corresponding component or loss term modified in each setting. All ablation experiments are evaluated on the degraded-only subset of the held-out test set.

#### 4.3.1 Component analysis of dynamic prompt generation and fusion.

To assess the contributions of the degradation predictor, prompt bank, and prompt-fusion mechanism, we design four component configurations for the ablation experiments, with the results presented in Table 3. In the shared-static-prompt setting, the degradation predictor and prompt bank are completely removed, and only a learnable shared static prompt vector is retained; therefore, the model cannot adaptively adjust degradation prompts according to the input image. The uniform weights setting retains the prompt bank but removes the degradation predictor, assigning equal weights to all degradation prompts, with the weighting vector defined as α = [1/K,···, 1/K], to evaluate restoration performance in the absence of degradation-aware weighting. In the without-prompt-fusion setting, the degradation predictor and prompt bank are retained, whereas the prompt-fusion mechanism is removed, preventing the generated dynamic prompt from modulating the restoration features. The full model uses the softmax probabilities predicted by the degradation predictor as dynamic prompt weights and integrates the resulting prompt into the restoration features through prompt fusion.

**Table 3:** Component ablation results for dynamic prompt generation and fusion.

Model	Predictor	Prompt bank	Prompt fusion	Prompt strategy	PSNR↑	SSIM↑
Shared static prompt	—	—	✓	Learnable static prompt	36.1410	0.9569
Uniform weights	—	✓	✓	α = [1/K, …, 1/K]	35.9761	0.9575
w/o prompt fusion	✓	✓	—	α = softmax(z)	35.7530	0.9561
Full model	✓	✓	✓	α = softmax(z)	**37.4890**	**0.9631**

Table 3 shows that the full model obtains the best PSNR and SSIM values, reaching 37.4890 dB and 0.9631, respectively. The shared-static-prompt and uniform-weights settings show similar performance, suggesting that simply incorporating a prompt bank or adopting a fixed shared prompt is not sufficient to fully capture differences among degradation types. In addition, removing the prompt-fusion mechanism reduces the PSNR and SSIM values to 35.7530 dB and 0.9561, respectively, indicating that the dynamically generated prompts need to be effectively integrated into the restoration features. By contrast, the full model produces input-dependent dynamic prompt weights through the degradation predictor and further incorporates the resulting prompt into the restoration features through prompt fusion, enabling the restoration network to perform degradation-adaptive restoration more effectively and thereby improving overall restoration performance.

#### 4.3.2 Number of PFE blocks.

The proposed progressive prompt fusion enhancement module consists of multiple PFE blocks; therefore, we further examine the influence of the number of PFE blocks on restoration performance. For this analysis, the number of PFE blocks is set to 1, 3, and 5, respectively, while all other training configurations are kept unchanged. The experimental results are presented in Table 4.

**Table 4:** Effect of the number of PFE blocks on restoration performance for degraded samples.

Model	Number of blocks	PSNR↑	SSIM↑	LPIPS↓
TIR-UPF	1	35.3942	0.9566	0.0818
	3	**37.4890**	**0.9631**	**0.0651**
	5	36.0660	0.9610	0.0676

Table 4 shows that increasing the number of PFE blocks from 1 to 3 improves restoration performance, suggesting that progressive prompt fusion strengthens the model’s degradation-adaptive restoration capability under different weather conditions. However, increasing the number of PFE blocks further to 5 does not yield additional performance improvements and may increase model complexity. Therefore, 3 PFE blocks are adopted as the default configuration in the proposed model.

#### 4.3.3 Ablation study on the joint loss function.

As shown in Table 5, incorporating the edge loss enables Group B to outperform Group A across all three metrics, demonstrating the effectiveness of the edge constraint in preserving structural details. Adding either the degradation-aware classification loss *L*_cls_ or the prompt orthogonality regularization *L*_ortho_ individually improves SSIM and LPIPS, although a decrease in PSNR is observed. When the two auxiliary terms are jointly employed, Group E achieves the best overall performance, with a PSNR of 37.4890 dB, an SSIM of 0.9631, and an LPIPS of 0.0651. These results indicate that the two auxiliary constraints are complementary and jointly promote a better balance among pixel-level fidelity, structural preservation, and perceptual quality.

**Table 5:** Ablation results of different loss settings on the degraded-only test subset.

Group	Loss setting	*L*_*char*_	*L*_*edge*_	*L*_*cls*_	*L*_*ortho*_	PSNR↑	SSIM↑	LPIPS↓
A	Base loss	✓	—	—	—	35.6689	0.9551	0.0837
B	+Edge	✓	✓	—	—	36.8769	0.9575	0.0781
C	w/o ***L***_***ortho***_	✓	✓	✓	—	36.1237	0.9610	0.0683
D	w/o ***L***_***cls***_	✓	✓	—	✓	36.2594	0.9591	0.0735
E	Full loss	✓	✓	✓	✓	**37.4890**	**0.9631**	**0.0651**

#### 4.3.4 Statistical reliability under multiple random seeds.

To assess the robustness of the reported improvements to stochastic training variation, we repeated the complete TIR-UPF model, the Restormer baseline, and the variant without prompt fusion using three fixed random seeds (100, 1234, and 2025). Restormer was selected as the representative key baseline because it serves as the restoration backbone of TIR-UPF, while the variant without prompt fusion was selected as the critical controlled ablation for evaluating the proposed prompt-guided feature modulation. Across all independent runs, the training, validation, and test splits, degradation-generation pipeline, training schedule, hyperparameters, checkpoint-selection criterion, hardware environment, and evaluation protocol were kept unchanged. Table 6 reports the mean and sample standard deviation of the metrics on the degraded-only test subset (rain, snow, and haze images only) over the three independent training runs; thus, the reported standard deviations reflect variation across training runs rather than variation among individual test images. In contrast, the aggregate results in Table 2 are calculated over the complete test set of 840 images, including clean images.

**Table 6:** Statistical reliability on the degraded-only test subset across three independent training runs using random seeds 100, 1234, and 2025. Values are reported as mean ± sample standard deviation.

Method	PSNR↑	SSIM↑	LPIPS↓
Restormer	36.7232 ± 0.5765	0.9599 ± 0.0010	0.0932 ± 0.0124
TIR-UPF w/o Prompt Fusion	35.7772 ± 0.0216	0.9547 ± 0.0055	0.0766 ± 0.0081
TIR-UPF	**37.4890 ± 0.2388**	**0.9631 ± 0.0027**	**0.0651 ± 0.0076**

As shown in Table 6, TIR-UPF achieves the best average performance, with a PSNR of 37.4890 dB, an SSIM of 0.9631, and an LPIPS of 0.0651. Compared with Restormer, it improves PSNR and SSIM by 0.7658 dB and 0.0032, respectively, while reducing LPIPS by 0.0281. Compared with the variant without prompt fusion, it improves PSNR and SSIM by 1.7118 dB and 0.0084, respectively, and reduces LPIPS by 0.0115. Moreover, TIR-UPF outperformed both comparison methods under each of the three random seeds for all evaluated metrics, demonstrating consistent improvements across the repeated runs and supporting the effectiveness of the proposed prompt-fusion mechanism. This multi-seed experiment serves as a robustness analysis of the proposed method and representative comparisons, rather than a formal statistical significance analysis across all competing methods.

### 4.4 Downstream task experiments

This section evaluates the effectiveness of the proposed method using a downstream object detection task. A fixed YOLOv11x detector [39] with the official M3FD pretrained checkpoint M3FD-yolo11x-e300-8-pretrained-.pt is used for evaluation. During the downstream evaluation, the detector parameters are kept fixed, and no fine-tuning is performed on clean, degraded, or restored images. The detector is neither fine-tuned separately for different restoration methods nor jointly trained with the restoration network. All restoration methods are evaluated using the same detector checkpoint. The detector checkpoint and annotations are directly adopted from the official M3FD resources and are kept identical for all restoration methods. Therefore, this experiment is intended to provide a controlled relative comparison of the effects of different restoration methods on downstream detection rather than to evaluate the generalization ability of the detector itself.

The evaluation is conducted on the same 840 test images and corresponding annotations. The detector uses an input size of 640, a confidence threshold of 0.001, and an NMS IoU threshold of 0.6. Considering the imbalanced category distribution in the test set, the detection results are mainly reported for three major categories: person, car, and motorcycle. AP, mAP@0.5, mAP@0.5:0.95, and precision are computed based on these three categories using the Ultralytics validation procedure. Clean IR is provided as a reference result and is excluded from the method ranking, whereas degraded IR refers to detection performed directly on degraded infrared images.

As shown in Table 7, degraded images substantially decrease detection performance, with degraded IR reaching only mAP@0.5 = 0.3257 and mAP@0.5:0.95 = 0.1606. After restoration, all methods improve detection performance to different extents. The proposed method achieves the best overall detection performance, reaching mAP@0.5 = 0.5200 and mAP@0.5:0.95 = 0.2600. Compared with degraded IR, these results represent improvements of 19.43 and 9.94 percentage points, respectively. For category-wise AP, the proposed method obtains the best results for person, car, and motorcycle, indicating its ability to recover target information that benefits object detection. Compared with the clean IR reference, the proposed method achieves comparable overall detection performance.

**Table 7:** Quantitative comparison of different methods for the object detection task. Clean IR is provided as a reference result and is excluded from the method ranking.

Method	Person	Car	Motorcycle	mAP@0.5	mAP@0.5:0.95	Precision
Clean IR	0.4362	0.7401	0.3737	0.5167	0.2597	0.6962
Degraded	0.3115	0.5000	0.1655	0.3257	0.1606	0.5949
Restormer	0.4395	0.7370	0.3400	0.5055	0.2546	0.7218
AirNet	0.4088	0.7084	0.2759	0.4644	0.2310	0.6765
PromptIR	0.4308	0.7260	0.3415	0.4994	0.2505	0.6489
AdaIR	0.4398	0.7408	0.3592	0.5133	0.2595	0.7332
AWFusion	0.4322	0.7300	0.3216	0.4946	0.2488	0.6456
Proposed	**0.4401**	**0.7410**	**0.3789**	**0.5200**	**0.2600**	0.7667

### 4.5 Computational complexity analysis

All methods were evaluated in FP32 using an input resolution of 1024 × 768 and a batch size of 1 on the same NVIDIA GeForce RTX 5070 Ti GPU with 16 GB memory. FLOPs were calculated for a single forward pass, and inference time and peak GPU memory usage were measured under the same evaluation settings.

As shown in Table 8, TIR-UPF contains 26.63 M parameters, which is comparable to Restormer and lower than PromptIR, AdaIR, and AWFusion. Owing to its three-stage progressive restoration architecture, TIR-UPF requires 11171.79 G FLOPs and an average inference time of 2977.96 ms per image, indicating higher computational cost than single-stage methods such as Restormer, PromptIR, and AdaIR. Nevertheless, its peak GPU memory usage is 6.74 GB, which is comparable to Restormer and AdaIR and substantially lower than AWFusion. These results show that the improved restoration performance of TIR-UPF is obtained at the cost of increased computation and inference latency, while maintaining moderate parameter count and GPU memory consumption.

**Table 8:** Computational complexity comparison of different methods.

Method	Params (M)	FLOPs (G)	Inference time (ms/image)	Peak GPU memory (GB)
Restormer	26.13	**3727.00**	**936.37**	6.75
AirNet	**8.93**	10141.76	2845.98	**3.78**
PromptIR	35.59	4155.25	1015.56	6.86
AdaIR	28.76	3886.82	1044.73	6.75
AWFusion	50.47	21438.68	53348.42	11.87
Proposed	26.63	11171.79	2977.96	6.74

## 5. Conclusions

In this study, we introduce TIR-UPF, a unified progressive fusion network developed for thermal infrared image restoration under individually occurring rain, snow, or haze degradations. The proposed framework uses a lightweight degradation predictor to estimate the degradation-state distribution of an input thermal infrared image and generate degradation-aware prompts from a learnable prompt bank. These dynamic prompts are then embedded into the PFE module, enabling adaptive modulation of restoration features in the presence of rain, snow, and haze. Moreover, a joint loss function is formulated by integrating reconstruction loss, edge loss, and degradation-aware auxiliary constraints, which helps improve pixel-level accuracy, retain structural details, and strengthen the separability of prompt representations. Experimental findings demonstrate that TIR-UPF delivers competitive and generally superior performance compared with representative restoration approaches. Downstream object detection experiments further support its practical value for perception tasks in adverse weather environments. Overall, these results confirm the effectiveness of adaptive degradation prompts and PFE for unified thermal infrared image restoration across the evaluated weather degradation categories on the proposed M3FD-based synthetic benchmark and indicate the potential of the proposed framework as an image preprocessing component for autonomous driving under adverse-weather conditions, intelligent transportation, thermal surveillance, unmanned aerial sensing, mobile robotics, and search-and-rescue systems.

As the current evaluation is limited to individual weather degradations, and challenging cases may still result in incomplete restoration under certain conditions, future work will investigate the performance of the proposed framework under unseen compound degradation scenarios and on real-world adverse-weather TIR data.
